# Severe and Fatal Fentanyl Poisonings from Transdermal Systems after On-Skin and Ingestion Application

**DOI:** 10.3390/toxics11100872

**Published:** 2023-10-20

**Authors:** Karina Sommerfeld-Klatta, Wiktoria Jiers, Magdalena Łukasik-Głębocka, Artur Tezyk, Klaudia Dolińska-Kaczmarek, Kamil Walter, Paweł Świderski, Szymon Rzepczyk, Barbara Zielińska-Psuja, Czesław Żaba

**Affiliations:** 1 Department of Toxicology, Poznań University of Medical Sciences, 30 Dojazd Street, 60-631 Poznan, Poland; 2 Department of Emergency Medicine, Poznań University of Medical Sciences, 7 Rokietnicka Street, 60-806 Poznan, Poland; 3 Department of Forensic Medicine, Poznań University of Medical Sciences, 10 Rokietnicka Street, 60-806 Poznan, Poland

**Keywords:** fentanyl, poisoning, transdermal, ingestion

## Abstract

In recent years, the administration of fentanyl (FNTL) implicitly in transdermal drug delivery systems (TDDS) has vastly increased in chronic pain treatment. Non-medical and uncontrolled use of FNTL in TFDS (transdermal fentanyl delivery systems) may reveal toxic effects by the route of exposure, dermal or alternative, by ingestion of patches, and drug release in the stomach. The purpose of this study was to present three different cases of FNTL poisonings, two of which resulted in death due to TFDS abuse. The first case is a 66-year-old woman treated for accidental FTNL poisoning resulting in acute respiratory distress syndrome. Two remaining cases are a 31-year-old woman and a 25-year-old man who died as a result of FNTL overdose after on-skin and ingestion application of the drug patches. During the hospitalization of the 66-year-old patient, in blood samples, FNTL was confirmed at a concentration of 10.0 ng/mL. Tests run on blood taken from the corpses of 25- and 31-year-old patients exhibited FNTL presence in concentrations of 29.1 ng/mL and 38.7 ng/mL, respectively. The various routes of administration and ultimately toxic effects are important to present because, in TDDS, fentanyl can be a reason for severe to fatal poisoning, as shown by the three cases above.

## 1. Introduction

One of the key challenges of modern medicine is effective pain treatment, which involves improving the quality of life of patients. Opioid drugs have become significantly more popular in the treatment of chronic pain in recent years, becoming more available and also in the illegal trade [1,2,3,4]. Fentanyl (FNTL) is a strong opioid (approximately 100 times stronger than morphine) with affinity for µ-opioid receptors. It was first synthesized in the 1950s. And was introduced into clinical use in 1963. Currently, it is a medicine available only by prescription [5,6].

**Figure 1:**
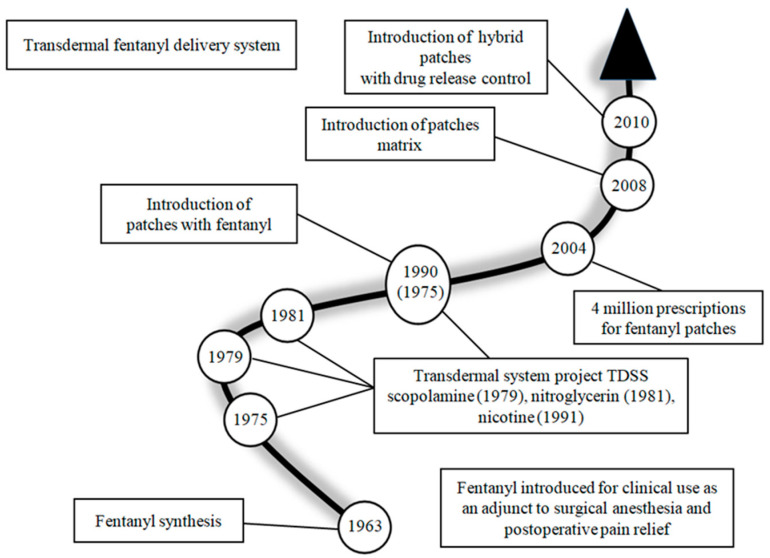
History of the introduction of TDDS and the evolution of this route of administration [7,10,11,12,13].

Formulations containing FNTL are injections (intravenous, intramuscular, subcutaneous, and sublingual), nasal sprays, sublingual tablets, and transdermal drug delivery systems (TDDS) [4,6]. The latter provide great support in the mitigation of pain, improving the patient’s quality of life, and ensuring the convenience of using the selected drug in the form of patches stuck to the skin. In this way, the substance administered (transdermally) bypasses the gastrointestinal tract and is not broken down, and thus a therapeutic effect is achieved even at lower doses than after oral administration [6,7,8,9]. The first transdermal systems were introduced into medical treatment in the USA. These were TTS (transdermal therapeutic systems) with scopolamine used for motion sickness and with nitroglycerin used for coronary heart disease. Nowadays, TTS is widespread in many areas of medicine. The history of the introduction of TDDS and the evolution of this route of administration is presented in Figure 1 [7,10,11,12,13].

**Figure 2:**
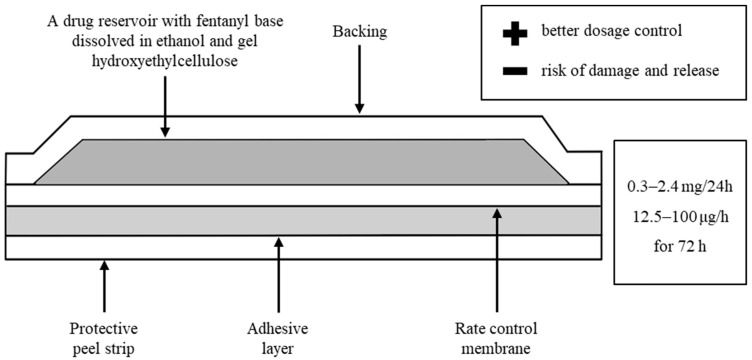
TDDS consists of three permanent elements: a reservoir of the active substance, an element controlling the release (matrix, membrane), and an energy source [10,14].

TDDS releases the drug at a programmed, constant rate for a set period of time. The patch consists of three solid elements: a reservoir of the active substance, a release control element (matrix, membrane), and an energy source (mechanical energy, electrical energy, osmotic pressure, and concentration gradient) (Figure 2) [10,14].

The increased popularity of opioid analgesics has resulted in an increase in the number of patients addicted to painkillers in this category, as well as the development of illegal trade in opioid substances. Illegal fentanyl can be obtained in forms other than those available on prescription, e.g., powder or enteric-coated tablets, as well as mixed with other substances, e.g., heroin or cocaine [8].

Fentanyl is a lipophilic drug that penetrates the blood-brain barrier well. It strongly stimulates the vomiting center while inhibiting the respiratory center [1,15]. Fentanyl is metabolized mainly in the liver by the cytochrome P450 3A4 isoenzyme system (CYP450). It undergoes rapid and extensive first-pass metabolism through oxidative N-dealkylation to norfentanyl (NOR) and other inactive metabolites [5]. Various TFDS formulations containing FNTL at different doses are available on the pharmaceutical market, e.g., 12 µg/h drug-releasing formulations containing 1.38 mg of fentanyl in a 4.2 cm^2^ patch or 100 µg/h containing 11 mg of drug in a 33.6 cm^2^ patch [7]. Importantly, fentanyl concentration and its effects depend on the form of its administration (Table 1) [1,5]. In the case of transdermal systems, the maximum concentration ranges from 0.3 ng/mL for 12 µg/h to 2.6 ng/mL for 100 µg/h, where the drug is released gradually over 12–24 h, and in the following hours the concentrations remain relatively constant. The therapeutic analgesic concentration of FNTL in the blood ranges from 1 to 4 ng/mL, and standard patch treatment begins with doses of 12–25 µg/h in patients who have previously used weak opioids (e.g., tramadol). The dosage is individual for each patient and takes into account: the history, the possibility of developing tolerance, the patient’s general health condition and clinical condition, as well as the severity of the disease [1,16,17,18,19].

**Figure 3:**
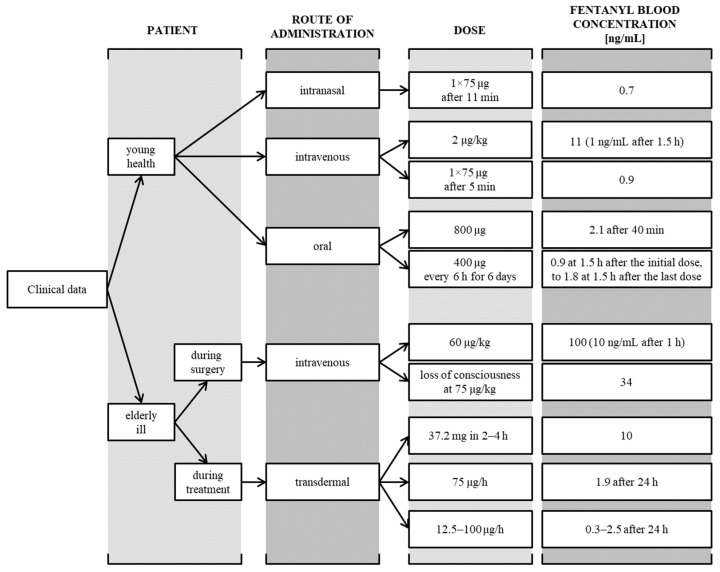
Clinical data on fentanyl exposure and acute poisoning [14,17,18,20,21,22,23,24].

**Figure 4:**
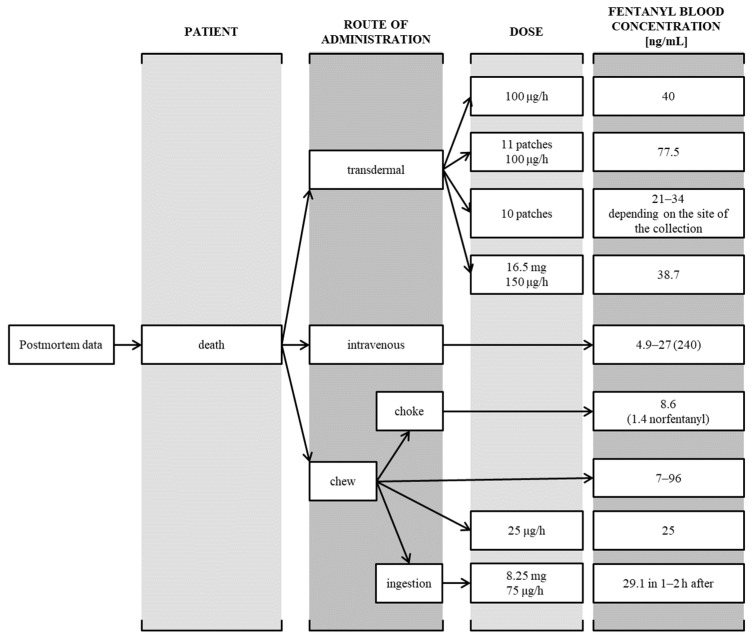
Postmortem data on fentanyl fatal poisoning [25,26,27,28,29,30,31,32].

Fentanyl, as a strong opioid, requires special precautions during use due to possible interactions and associated side effects; therefore, selecting the appropriate dosage is crucial to avoid acute poisoning (Figure 3) [14,17,18,19,20,21,22,23,24]. Moreover, in itself, it can cause, among others, respiratory failure, a life-threatening condition. The main toxic effect of fentanyl is respiratory depression. Respiratory failure may be accompanied by, among others: cyanosis, Cheyne-Stokes breathing, hypotonia, hypothermia, bradycardia, and hypotension; moreover, the lethal dose depends on the route of administration of the substance (Figure 4) [25,26,27,28,29,30,31,32]. Additionally, convulsions, deep sedation, miosis, ataxia, and—particularly in pediatric patients—fever, vomiting, and nausea may occur [1,15]. The mainstay of treatment for fentanyl poisoning given as TFDS is the removal of the transdermal patch. The antidote is an opioid receptor antagonist, i.e., naloxone. Management of adult patients includes the administration of naloxone hydrochloride i.v. in a dose ranging from 0.4 to 2 mg. The dose should be repeated every 2–3 min until the clinical condition of the patient improves. An alternative is an intravenous infusion of 2 mg in 500 mL of 0.9% NaCl or 2 mg in a 50 mg/L (5%) glucose solution. With the exception of antidote use, the patient’s vital signs should be monitored. It is necessary to maintain airway patency. The patient’s body temperature should be maintained, and fluids should be adequately supplied [13,14]. The aim of the study was to describe cases of fentanyl poisoning from transdermal systems after on-skin and ingestion application.

## 2. Case Reports

### 2.1. Case 1

A 66-year-old female patient was admitted to the Department of Toxicology with signs of acute cardiopulmonary failure and acute kidney and liver damage. The patient had a medical history of pulmonary embolism with cardiogenic shock and acute liver and kidney failure two years earlier, type II diabetes, arterial hypertension, back pain syndrome, depression, and obesity. Due to spine pain, the patient was administered FNTL patches twice: three patches containing fentanyl at a dose of 75 µg/h, 4 days before admission to the hospital, and one patch at the same dose the day before. The patient with signs of shock was transported to the hospital emergency department, and from there—after her condition was stabilized—she was transferred to the Department of Toxicology. Symptoms of poisoning (psychomotor retardation, hypotension, and respiratory depression) persisted for 5 days. During this time, the patient received a continuous infusion of Naloxone and Levonor, and due to increased inflammatory parameters (C-reactive protein, CRP 56.2 mg/L), empirical antibiotic therapy was started (Biotraxon at a dose of 1 g i.v.). The patient was also given HNF (fractionated heparin) on the pump due to a non-excludable pulmonary embolism (history) and Furosemide (also on the pump) to achieve diuresis. After 5 days of treatment, the patient’s clinical condition improved, and the treatment was discontinued. After a total of 11 days of hospitalization, the patient was discharged. Blood was collected for toxicological tests three times, 2, 6, and 12 h after admission to the department, in which fentanyl and its metabolite, norfentanyl, were determined.

### 2.2. Case 2

A 31-year-old woman was found unconscious by her mother at around 3 a.m. After calling an ambulance and 20 min of resuscitation, she was transported to the hospital emergency department (after about 15 min). In the hospital, another resuscitation was performed lasting 30 min, and within the next 30 min there was a change in the rhythm: from ventricular fibrillation to PEA (pulseless electrical activity), and then asystole, where death was declared. The patient abused FNTL in the form of patches that released 50 µg/h of the drug in a preparation containing 5.5 mg of FNTL. The patches belonged to the patient’s grandmother and were stuck to the lumbar region. The woman left a letter, which indicated that it was a suicide. Post-mortem concentrations of fentanyl and norfentanyl in blood were determined.

### 2.3. Case 3

A 25-year-old man was found dead at home, with injuries on his forearm consistent with self-inflicted wounds, most likely a few hours after death. During the autopsy, a patch was found in the stomach, containing 8.25 mg of fentanyl in a preparation with a release rate of 75 µg/h. The patch was swallowed whole and was not damaged. It was not determined where the man obtained the drug or whether he had used it before. Blood and urine were collected from the corpse, and the concentrations of the drug and metabolite were determined.

### 2.4. Fentanyl and Norfentanyl Analysis

Blood and urine were analyzed by high-pressure liquid chromatography using an Agilent 6410B QQQ series (Waldbronn, Germany) coupled to a triple quadrupole mass spectrometer (Table 2). A gradient program was used—90% [A] and 10% [B] for 1 min; 60% [A] and 40% [B] in 5 min; 60% [A] and 40% [B] for 2 min; 90% [A] and 10% [B] for 2 min; 90% [A] and 10% [B]. An amount of 100 µL of the internal standard, fentanyl D5, was added to 200 µL of blood/urine by performing liquid-liquid extraction with chlorobutane (1 mL) in a saturated sodium tetraborate solution at pH 9.8 (200 µL). Samples were shaken (3 min) and centrifuged (8 min/18,000 rpm). The organic layer was then evaporated, and the dry residue was dissolved in 100 μL of 0.1% formic acid with acetonitrile.

### 2.5. Results

In the case of a 66-year-old woman, during hospitalization, it was decided to perform toxicological analysis of the blood after 2, 6, and 12 h, respectively, using the liquid chromatography method (LC-MS/MS). The results were 10.0 ng/mL, 1.2 ng/mL, and <1 ng/mL, respectively. In the case of a 31-year-old woman, the FNTL concentration in her blood was found to be 38.7 ng/mL, and the NOR concentration was 149 ng/mL. In a 25-year-old man, FNTL and NOR were found in the blood at concentrations of 29.1 and 12.9 ng/mL and 4.5 and 112.0 ng/mL in the urine. No other drugs were found. A comparison of the concentrations obtained is presented in Table 3.

A comparison of the dose, method of application, and source of the drug is presented in Table 4.

## 3. Discussion

Opioid analgesics differ in their way of interacting with receptors, distribution, elimination routes, and half-life. Therefore, it is necessary to choose the preparation and dose wisely, depending on the individual needs of the patient [1,2,3]. An overdose of fentanyl can result in respiratory failure and death. Children are particularly at risk of accidental fentanyl overdose, and attention should also be paid to exposure resulting from patch ingestion. Another factor in overdose is mental disorders and addiction to psychoactive substances. People who use opioids for purposes other than pain management are highly aware of their risks [4,19].

This leads the authors of the study to conclude that a fentanyl overdose is often not the result of a lack of knowledge and awareness of the risk, but on the contrary—people with mental disorders and addictions use the increasingly available knowledge about the dangers associated with high concentrations of opioid drugs and use them to achieve their own goals (e.g., to commit suicide or simply become intoxicated). A major problem in the context of overdoses of opioid drugs, including fentanyl, is the increased availability of these substances, especially on the black market. According to official data, pure fentanyl for intravenous administration is rarely available from illegal sources because it is used only in inpatient treatment. The most common products on the market are preparations with the addition of opioids, e.g., powders and tablets [6,9]. Prescription drugs, in addition to illegal purchases, come from, for example, family members. This is important information in terms of the risk of poisoning. It is easy to calculate that the smallest patch containing 1250 µg of fentanyl is 10–20 times more than the therapeutic dose intravenously of 50–100 µg (1 µg/kg) [11]. In addition to the dose, the drug’s pharmacokinetics also influence the course of poisoning, which varies depending on the route of administration (Figure 3 and Figure 4). This information indicates that the use of patches carries a greater risk of poisoning than intravenous administration—this is due to both the better availability and higher concentration of the drug, as well as its much longer elimination from the body [14,16,21].

The literature describes cases of acute and fatal poisonings using patches containing fentanyl through various absorption routes [22,23,25,26,27,29,30,31,32]. The presented work presents a summary of three cases, including one acute poisoning caused by improper use of TFDS (case 1—the highest FNTL concentration in blood taken upon admission to the hospital was 10 ng/mL) and two fatal cases, one of which was a classic course resulting from suicide (case 2—FNTL concentration in autopsy blood 38.7 ng/mL) and the second one, which was distinguished from the others by the route of administration and provoked discussion on the toxicity of the drug in the form of ingestion of an intact patch from which the drug could be released at an inestimable rate in the stomach, causing death (case 3—FNTL concentration in autopsy blood 29.1 ng/mL) (Table 3 and Table 4).

TFDS poisoning is also discussed in the literature in the context of a mistake. Bakovic et al. presented the story of a 2-year-old girl for whom a fentanyl patch (25 µg/h) was applied to a knee wound. Toxicology analysis showed trace levels of fentanyl in the blood just above the detection limit (2 ng/mL), while concentrations in urine, liver, and kidney were approximately 102, 28, and 10 ng/mL, respectively. Applying the patch to broken, bleeding skin was associated with an increased rate of skin absorption of fentanyl [33]. Higher body water content in children, together with a larger volume of distribution and greater clearance than in adults, were associated with a significant reduction in the biological half-life of the drug and an increase in its toxicity. Similarly, in the case of a man who was treated with a fentanyl patch on damaged skin (12 µg/h) in the course of atopic dermatitis [34].

It is a fact that accidental fatal poisonings are rare, but it is very important to raise awareness about the dangers of misuse of TFDS, especially since the vast majority of them are preventable. Interestingly, single cases of poisoning with patches containing fentanyl via alternative routes, such as the alimentary or respiratory route, have been described. Moore et al. described the case of a 42-year-old man with lymphoma who was administered transdermal fentanyl therapy (150 µg/h) during hospitalization due to chronic abdominal pain. During hospitalization, apnea and cyanosis occurred, which were observed several dozen hours after applying the patch to the skin and probably after ingesting the patch through the oral route. During an attempt at resuscitation, a piece of the plaster was removed from the patient’s throat [35]. A few years earlier, Mrvos et al. presented a review of poisonings involving 76 cases of fentanyl patch consumption, 45 men and 31 women, with an average age of 33 years. The largest number of patches consumed was 5, but patients were most frequently exposed to 1 patch taken orally. The greatest percentage of patients presented with symptoms such as coma, drowsiness, and respiratory depression. In most cases, the time from ingestion to the onset of symptoms has not been determined. In the group in question, 2 people died. A total of 56 patients were treated in the intensive care unit. A total of 63 patients required the administration of naloxone, fluid therapy, and oxygen. Difficulties were also encountered in determining the dose, where the maximum was 100 µg/h [36]. As in the presented cases, the poisonings were the result of a mistake or a suicide attempt. For a full comparison of the presented cases by Mrvos, there were no results of fentanyl concentrations measured in blood during poisoning or in blood taken postmortem.

Similar conclusions were found regarding the easier absorption of fentanyl after swallowing and the more difficult removal of the patch from the body as a result of its alternative aspiration (from the respiratory tract or stomach). Interestingly, Carson et al. presented the story of a person who died as a result of the rare and inappropriate use of a fentanyl transdermal patch due to chewing, followed by complications following patch aspiration. The poisoned individual was a 28-year-old man with a medical history of prescription drug abuse who was pronounced dead in the emergency department shortly after admission. During the autopsy, a piece of the patch was identified that was stuck in the main bronchus. Toxicological analysis of the blood showed methamphetamine, fentanyl, and norfentanyl concentrations of 1456, 8.6, and 1.4 ng/mL, respectively [30].

Analyzing the results of fentanyl concentrations in the blood of a patient who was mistakenly poisoned with TFDS (75 µg/h) during the treatment of spinal pain (case No. 1), it can be concluded that they were at a toxic level (the maximum concentration of FNTL reaches 0.3 ng/mL for 12 µg/h to 2.6 ng/mL for 100 µg/h, where the drug was gradually released over 12–24 h), and the observed symptoms were consistent with the picture of severe TFDS poisoning. Three-time analysis of blood during hospitalization, 2, 6, and 12 h after admission to treatment and removal of the applied patches, showed rapid elimination of the drug, supported by effective treatment with naloxone, despite persistent symptoms of poisoning for 5 days (psychomotor retardation, hypotonia, respiratory depression). Discussing case No. 2, it was about typical fatal suicidal poisoning. The drug not belonging to the poisoned person, the suicide note, and the high concentrations of FNTL in blood taken after death confirm this picture. Comparing case No. 3 to other unusual poisonings, it should be stated that this was certainly a story that represented an alternative method of exposure to TFDS. Undoubtedly, the interpretation of the obtained results was quite difficult due to the rarely reported poisonings in which an intact patch was found in the stomach during the autopsy, which was considered the direct cause of death. As in the presented case, it should be considered that people abusing prescription drugs, such as fentanyl, often modify the route of administration from the recommended one. As the case of Carson and the presented evidence show, this choice can be fatal [30]. In 2000, Anderson and Muto reported 25 cases of fatal poisonings. Fentanyl was present in blood collected from the heart (*n* = 23) at corresponding concentrations ranging from 1.8 to 139 ng/mL, and in the case of femoral (peripheral) blood, concentrations (*n* = 13) ranged from 3.1 to 43 ng/mL [30].

In conclusion, it is worth mentioning the results of research conducted by Plasencia et al. in 2014 on the simulated in vitro release of fentanyl from patches (at a dose of 75 µg/h) in gastric and intestinal fluid. Fluid samples were collected at time zero and after 5, 15, 30, 60, 120, and 180 min of immersion. Fentanyl was determined using ultraperformance liquid chromatography combined with tandem mass spectrometry. An average of 239 µg and 1962 µg of fentanyl were released into the gastric fluid and 338 µg and 3139 µg into the intestinal fluid after 5 min and 3 h, respectively. An average of 26% and 41% of the 7.65 mg of fentanyl contained in the 75 µg/h patch were released into the gastric and intestinal fluids, respectively, within 3 h [37]. These results confirm a very rapid drug release process within a few minutes after immersion, which could be compared to the results presented in Case No. 3 (FNTL concentration in autopsy blood: 29.1 ng/mL), where FNTL was released from the intact patch already in the stomach after its consumption. Numerous mutilations revealed during the autopsy, in the absence of information about the man’s treatment or abuse of TFDS, allow one to conclude that it could have been a suicide using a patch containing FNTL ingested through the oral route, from which the drug was absorbed only in the stomach (no patches applied to the skin). In confirmation of the hypothesis, the presence of the drug and metabolite was also recorded in the deceased’s urine (FNTL concentration 4.5 ng/mL and NOR 112 ng/mL in urine). In addition, the reuse of medication patches intended for disposal is also an important issue. After use, such patches go to medical waste, where they can be taken out and then boiled to extract the remaining active substance. Therefore, it is important to properly and carefully manage treatment residues from healthcare staff.

## 4. Conclusions

A disturbing trend is the case of poisonings where fentanyl in the form of TDDS is used in an unusual way, through oral, intravenous, or respiratory routes. Additionally, chewing the patch may cause the drug to be absorbed not only in the stomach but also in the mouth. Although fentanyl is characterized by low bioavailability, in the event of oral poisoning, the active substance released from transdermal patches may lead to the patient’s death. Improper use of transdermal patches containing fentanyl poses a serious threat to the health and life of patients, which indicates the need for good education of patients and their families about the mode of action and the risk of poisoning.

## Figures and Tables

**Table 1:** Effects of FNTL after intravenous, TDDS, and oral administration [1,16,17,18,19].

Intravenous (i.v.)	TDDS	per os (p.o.)
After intravenous administration, fast effects (a few minutes), duration after administration of 100 µg up to 60 min	After transdermal administration directly into the circulation, bypassing the first pass effect, reduction of side effects	After oral administration (chewing and swallowing) of fentanyl patches, relatively low bioavailability (30–50%), toxicity may result from a relatively large dose of the drug in the patch
Quick redistribution to muscles, adipose tissue and then slowly to the bloodstream	Possible administration of the drug in a constant continuous dose for 3 days	There are known cases of acute/fatal poisoning after: ingestion or exposure through the respiratory tract (drinking the obtained extract or inhaling vapors when cooking fentanyl patches) or after ingesting an intact patch
Only a constant infusion ensures tissue saturation, drug accumulation and longer effects	The release rate depends on the size of the patch	
	Therapeutic concentration within 12 h, constant after 36–48 h	

**Table 2:** Method parameters.

Source of Ionisation	Electrospray (Agilent 6410B, Wildnington, DE, USA)
MS mode of operation	MRM, recording two reactions for each compound
Column	Poroshell 120 EC-18 3.0 × 75 mm, 2.7 μm (Agilent, USA)
Mobile phases	0.1% formic acid in water [A] and 0.1% formic acid in acetonitrile [B]
Flow rate	0.5 mL/min

**Table 3:** Obtained concentrations of fentanyl and norfentanyl in the presented cases.

Case No	Patient’s Sex	Age [Years]	Marking	Specimen Type	C_FNTL_ [ng/mL]	C_NOR_ [ng/mL]
1	Female	66	living, after 2, 6 and 12 h	blood	2 h: 10.0 6 h: 1.2 12 h: <1	2 h: 49.7 6 h: 31.2 12 h: 16.1
2	Female	31	posthumous	blood	38.7	149.0
3	Male	25	posthumous	blood and urine	29.1 in blood 4.5 in urine	12.9 in blood 112.0 in urine

**Table 4:** Dose, method, time of application, and source of drug.

Case No	Dose of Drug per Patch [µg/h]	Method of Application	Duration of Use	Source
1	75	on skin	4 days	own drug
2	50	on skin	probably several or several dozen hours	family member’s drug
3	75	swallowed, in the stomach	probably a few hours	no data

## Data Availability

The data presented in this study are available upon request from the corresponding authors.
